# Young convalescent COVID‐19 pneumonia with extensive pneumomediastinum emphysema: Case report

**DOI:** 10.1002/ccr3.5543

**Published:** 2022-03-27

**Authors:** Marta Jagosz, Wiktoria Guzik, Łukasz Moczała, Mateusz Rydel, Hanna Misiołek, Szymon Białka

**Affiliations:** ^1^ Faculty of Medical Sciences in Zabrze Student Scientific Society at the Department of Anaesthesiology, Intensive Care and Emergency Medicine Medical University of Silesia Katowice Poland; ^2^ Faculty of Medical Sciences in Zabrze, Chair and Department of Thoracic Surgery Medical University of Silesia Katowice Poland; ^3^ Faculty of Medical Sciences in Zabrze Department of Anaesthesiology, Intensive Care and Emergency Medicine Medical University of Silesia Katowice Poland

**Keywords:** co‐ARDS, COVID‐19 pneumonia, pneumothorax, spontaneous pneumomediastinum (SP), subcutaneous emphysema

## Abstract

The development of the SARS‐CoV‐2 pandemic caused a common appearance of severe pulmonary complications, rarely seen as a result of the other infections. These are pneumothorax, pneumomediastinum, emphysematous bullae, cavitary lung lesions, or subcutaneous emphysema. Their formation is influenced by both—the natural course of the disease and the treatment strategy adopted.

## Graphical abstract

Development of SARS‐CoV‐2 severe pulmonary complications is influenced by both‐the natural course of the disease and the treatment strategy adopted. Appropriate, non‐aggressive ventilation and adequate treatment is essential for COVID‐19 convalescents and may prevent them from implementing invasive surgical procedures.

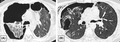

## 1 INTRODUCTION

Since December 2019, a coronavirus disease 2019 (COVID‐19) caused by severe acute respiratory infection with coronavirus 2 (SARS‐CoV‐2) has been spreading worldwide, resulting in a global pandemic.^1^ The most prevalent clinical features of COVID‐19 are fever, myalgia, shortness of breath, and cough. The diagnosis is made by real‐time–reverse transcription polymerase chain reaction (rRT‐PCR) from a nasopharyngeal swab.^2^ CT scans are essential to evaluate and to follow up the suspected COVID‐19 cases. The main CT feature of COVID‐19 pneumonia is the bilateral vast ground‐glass opacification (GGO). Baratella et al. reported that in the short‐term follow‐up of the SARS‐CoV‐2‐related ARDS survivors, the most frequent CT alterations were linear bands, GGOs, reticulations, bronchiolectasis, consolidations, bronchiectasis, and volume loss, respectively. They have recorded a reticular pattern with a posterior distribution in short‐term follow‐up which might be associated with the use of systematic corticosteroid.^3^ Pleural effusion, pericardial effusion, or pneumothorax is less frequently reported throughout the course of the disease.^4^ Pneumomediastinum, as a relatively rare presentation of COVID‐19 pneumonia, can occur in both non‐mechanical ventilated and ventilated patients. It might be the result of spreading inflammation in the lungs along with the change in their architecture.^5^ Baratella et al. have hypothesized that sonic hedgehog (SHH) and Wnt5a signaling might have been associated with COVID‐19 pneumomediastinum tracheal lesions as the autopsies of COVID‐19 cases showed an irregular regenerative process with cartilage tissue remodeling (unpresented in non‐COVID‐19 cases).^6^

The course of COVID‐19 may take various forms from asymptomatic to severe respiratory failure. The severity of the disease is influenced by many, not fully diagnosed factors. As a result, even young, previously healthy people can develop acute respiratory distress syndrome (ARDS) in the course of COVID‐19.

We report the case of a young convalescent COVID‐19 pneumonia with extensive pneumomediastinum emphysema and acute respiratory distress syndrome.

## 2 CASE REPORT

A 30‐year‐old male patient in critical condition with acute respiratory failure in the course of viral pneumonia of COVID‐19 etiology without any comorbidities and with no smoking history was transferred from the Department of Anaesthesiology and Intensive Care, Infectious Disease Hospital to the intensive care unit in our clinic to perform the thoracic surgery (bullectomy) procedure.

**FIGURE 1:**
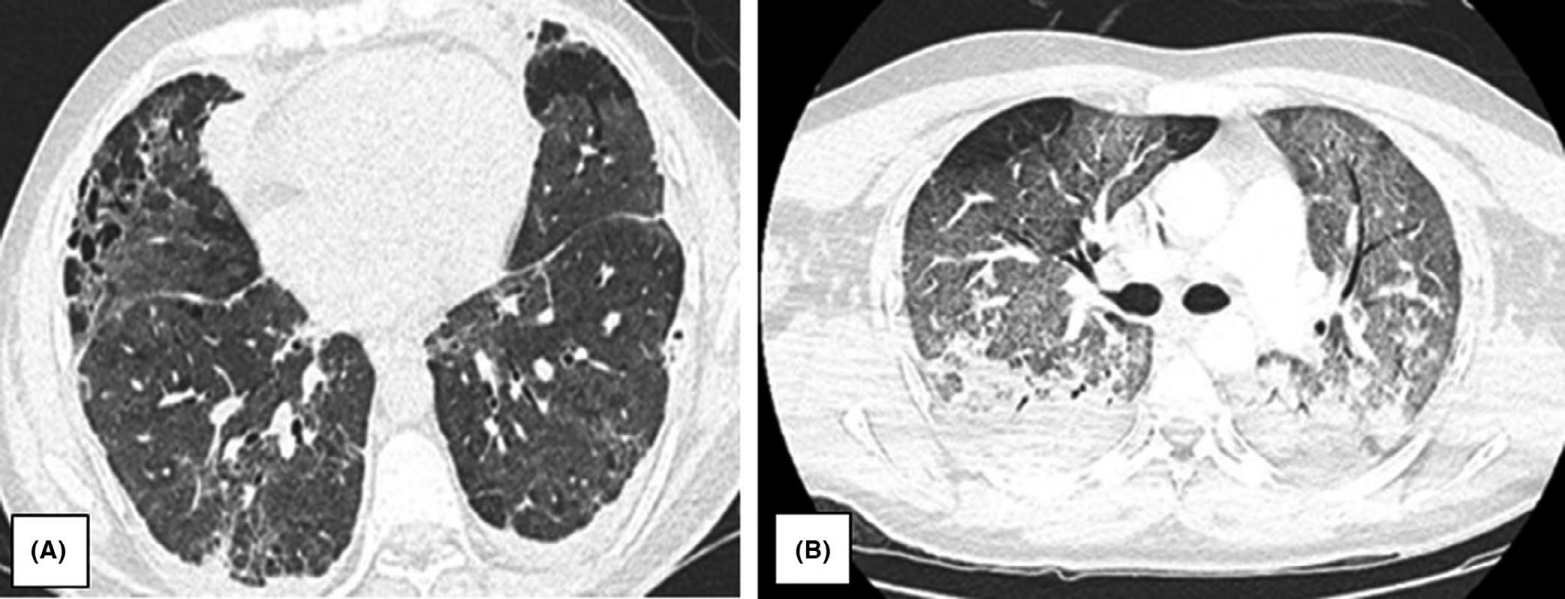
(A) Mild ground‐glass opacities. (B) Ground‐glass opacities, septal thickening, consolidations, and pleural effusions

The patient presented acute respiratory distress syndrome, mediastinal and right pleuritic pneumothorax, emphysematous bullae, and cavitary lung lesions with subcutaneous emphysema. Due to pleural effusion, the patient had an underwater‐sealed drain (UWSD) in both pleural cavities. CT scan was performed showing post‐COVID‐19 alterations (Figure 1A,B).

The past medical record related to the coronavirus infection included pulmonary embolism, thrombus in the right ventricle, fluid in the pericardial sac, tricuspid and mitral valve regurgitation, acute renal failure, coinfection with *A. Baumanii*, *K. pneumoniae*, sepsis caused by the *S. epidermidis* MSSE, polyneuropathy, protein‐calorie malnutrition, and anemia. The real‐time polymerase chain reaction COVID‐19 test (RT PCR) was performed with a negative result. On admission, the patient was stable, conscious, responsive and alert with respiratory insufficiency, mechanically ventilated with tracheostomy tube in synchronized intermittent mechanical ventilation (SIMV) FIO2 0.5 mode. On physical examination, the blood pressure was 135/70 mmHg with the pulse rate of 120 beats per minute.

The antiarrhythmic, bronchodilatory, mucolytic, analgesic, prokinetic, and anticoagulant treatment was included. The targeted antibiotic therapy was continued. Enteral feeding and vitamin supplementation were initiated, and alkalosis and electrolyte imbalance was leveled.

**FIGURE 2:**
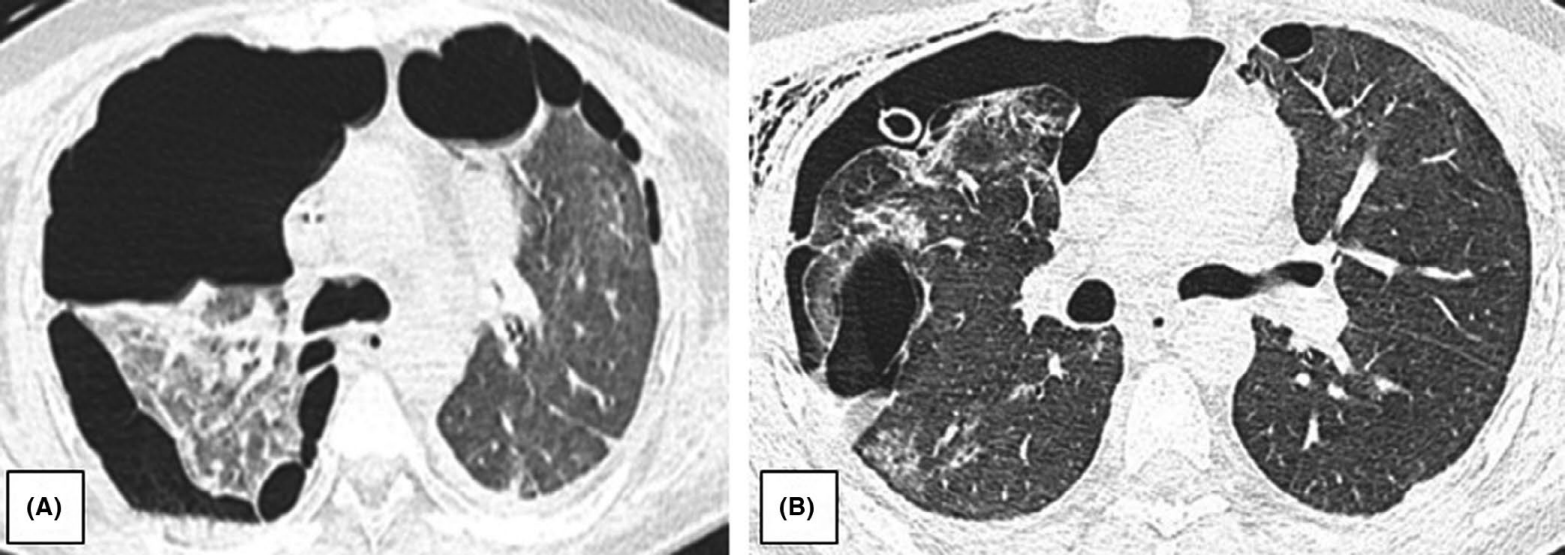
(A) Bilateral pneumothorax with the right lung collapse. (B) Pneumothorax drainage with the right lung partial expansion. Subcutaneous emphysema

Initially, the mechanical ventilation mode was modified, the CPAP was included, and after 2 days it was changed to PSV during 1 day. Then, CPAP ventilation and weaning from mechanical ventilation were continued until the fifth day of treatment when the patient started to breathe spontaneously. Initially, spontaneous breathing was conducted with the support of high flow oxygen therapy through a tracheostomy tube. Then, after improving the gasometric parameters, passive oxygenation was performed. On the seventh day after admission, following thoracic surgery consultation, the drain was removed from the left pleural cavity, and the right one was deposited. After the procedure, control CT scanning was performed (Figure 2B) and significant regression of pulmonary pleural changes and better aeration of the right lung were observed compared to the previous CT scan taken 17 days before (Figure 2B). It also showed meaningful regression of the fluid‐containing emphysematous bullae at the apical zone and fluid reservoir in the left pleural cavity. On the basis of the foregoing, it was decided to withdraw the patient from the surgical procedure.

On the twelfth day, the patient was transferred to the thoracic surgery ward in good condition, four‐limb moving, circulatory efficient with spontaneous breathing through a tracheostomy tube. During the stay at the thoracic surgery ward, intensive systemic rehabilitation and kinesiotherapy were performed. Gradually, the general condition of the patient improved, including the respiratory capacity, which enabled decannulation and then discharge from the hospital.

**FIGURE 3:**
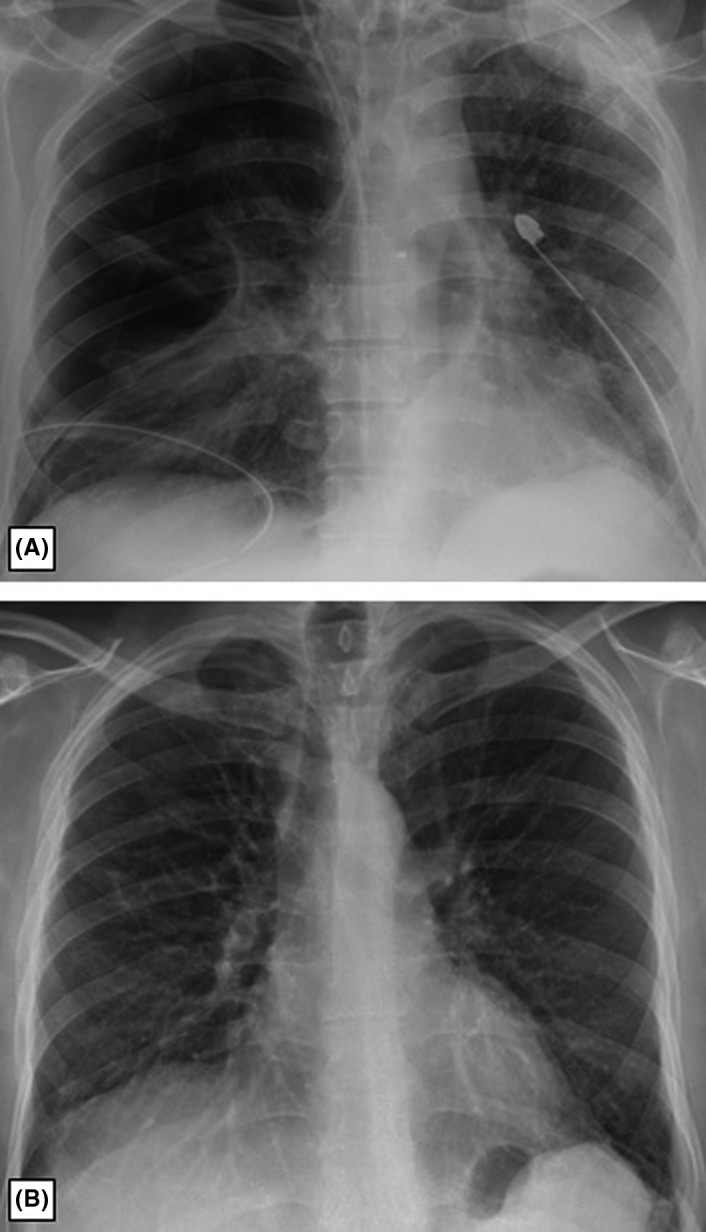
(A) Right pneumothorax with lung collapse. Bilateral chest drainage. (B) Right pneumothorax resolution after drainage with the complete right lung expansion

During 3 months of follow‐up, the patient was in general good condition and latest X‐ray (Figure 3B) has shown great regression of pulmonary changes compared to the X‐ray which was performed 3 months earlier (Figure 3A).

## 3 DISCUSSION

Multiple serious complications caused by COVID‐19 infection have been observed, where the most crucial and dangerous are the pulmonary ones. CT findings often show pneumothorax, spontaneous pneumomediastinum (SP), and subcutaneous emphysema. In the ICU, such complications usually appear in the course of ARDS, as an effect of barotrauma during aggressive mechanical ventilation; however, they are less frequent than in Co‐ARDS. That may indicate that patients after COVID‐19 show increased lung frailty and therefore require careful observation to prevent respiratory deterioration.^7^ Some studies show that even patients without invasive ventilation can develop pneumomediastinum and subcutaneous emphysema in the course of COVID‐19 pneumonia.^8^, ^9^

The rate of pulmonary embolus in the course of COVID‐19 is higher than normally recorded in patients without COVID‐19. Leonard‐ Lorant et al. show that 30% of patients with COVID‐19 infection were positive for acute pulmonary embolus. However, the median age of this cohort was 64+‐ 22.^10^ Pulmonary embolism risk factors associated with COVID‐19 do not contain traditional embolic risk factors. Anticoagulation before hospitalization, prophylactic anticoagulation during hospitalization, male gender, C‐reactive protein levels, and time from symptom onset to hospitalization are the factors associated with COVID‐19 pulmonary embolism.^11^ Mortality rate of COVID‐19 patients with pulmonary embolus was significantly greater than patients without PE regardless of other factors aggravating clinical condition of those patients. Frequency of cardiogenic shock was also significantly higher in PE cohort.^12^

To the best of our knowledge, this has been the first case report demonstrating this condition in such a young patient without any comorbidities.

## 4 CONCLUSIONS

The present case study shows that even very young patients without any comorbidities and primary changes in the lung parenchyma may develop serious complications after COVID‐19 infection. Appropriate, non‐aggressive ventilation and optimal treatment prevented further development and led to significant regression of the pulmonary changes. In consequence, the leakage on the drain was eliminated and the patient avoided the aggravating surgical procedure. Further studies and more clinical experience are needed to prevent this kind of condition in the present pandemic.

## CONFLICT OF INTEREST

None conflict of interest.

## AUTHOR CONTRIBUTIONS

MJ contributed to concept, data acquisition, and drafting article. WG and MR contributed to concept, data acquisition and analysis, and drafting article. LM contributed to data acquisition. HM contributed to critical revision of article, and final approval of the version to be published. SB contributed to concept, critical revision of article, and final approval of the version to be published.

## ETHICAL APPROVAL

There is no ethical concerns relating to this case report.

## CONSENT

Written informed consent was obtained from the patient to publish this report in accordance with the journal's patient consent policy.

## Data Availability

Data sharing is not applicable to this article as no new data were created or analyzed in this study.
